# Isolation and Functional Characterization of Soybean *BES1*/*BZR1* *Homolog* *3*-*Like* *1* (*GmBEH3L1*) Associated with Dehydration Sensitivity and Brassinosteroid Signaling in *Arabidopsis* *thaliana*

**DOI:** 10.3390/plants11192565

**Published:** 2022-09-29

**Authors:** Cho-Rong Park, Van Tinh Nguyen, Ji-Hee Min, Hyunkyu Sang, Gah-Hyun Lim, Cheol Soo Kim

**Affiliations:** 1 Department of Applied Biology, Chonnam National University, Gwangju 61186, Korea; 2 Department of Basic Science, Buon Ma Thuot University of Medicine and Pharmacy, Buon Ma Thuot 630000, Vietnam; 3 Department of Biochemistry and Biophysics, Texas A&M University, 300 Olsen Blvd, College Station, TX 77843-2128, USA; 4 Department of Integrative Food, Bioscience and Biotechnology, Chonnam National University, Gwangju 61186, Korea; 5 Department of Biological Sciences, Pusan National University, Busan 46241, Korea

**Keywords:** abscisic acid, BEH3 ortholog, brassinosteroid, osmotic stress, soybean GmBEH3L1

## Abstract

Brassinosteroid (BR) is an important steroid hormone that regulates plant development, abscisic acid (ABA) signaling, and responses to abiotic stress. We previously demonstrated that BEH3 (BES1/BZR1 Homolog 3) of *Arabidopsis* *thaliana* regulates dehydration and ABA responses by mediating proline metabolism. Furthermore, BEH3 negatively regulates BR-mediated hypocotyl elongation in dark-grown seedlings. However, the roles of *BEH3* ortholog genes in the osmotic stress response of plants have remained largely unknown. Here, *GmBEH3L1* (*Glycine max BEH3-Like 1*), a soybean (*G.* *max*) ortholog of the *BEH3* gene of *A. thaliana*, was isolated and functionally characterized. *GmBEH3L1* is induced by ABA, dehydration, and drought conditions. The *GmBEH3L1*-overexpressing transgenic lines (*GmBEH3L1*-OE/*beh3*) with the *beh3* mutant background have ABA- and dehydration-sensitive phenotypes during early seedling growth, implying that GmBEH3L1 is involved in both osmotic stress and ABA sensitivity as a negative regulator in *A. thaliana*. Consistent with these results, *GmBEH3L1*-OE/*beh3* complemental lines exhibit decreased expression levels of ABA- or dehydration-inducible genes. Under darkness, *GmBEH3L1*-OE/*beh3* complemental lines display a short hypocotyl length compared to the *beh3* mutant, indicating that GmBEH3L1 is linked to BR signaling. Together, our data suggest that GmBEH3L1 participates negatively in ABA and dehydration responses through BR signaling.

## 1. Introduction

Soybean, as a source of nutrients, oil, protein, and secondary metabolites in human diets, is one of the most important economic crops in the world. Drought is a major abiotic stress factor for plant growth and development, crop productivity, and biomass accumulation [1]. To improve soybean tolerance to drought stress, the identification of soybean’s molecular and physiological responses and phytohormone signaling regulatory networks are important [1,2,3].

In plants, dehydration stress increases the concentration of abscisic acid (ABA), an endogenous signaling regulator that initiates the adaptive response to water deficit stress [4,5]. In addition, plant development regulating hormones, brassinosteroids (BRs), also play a key role in drought stress [6,7]. Several studies described improved membrane stability, water uptake, nitrogen assimilation, and carbon dioxide rates in BR-treated compared to untreated plants under drought stress conditions [6,8,9]. However, Feng et al. [10] found that *Brachypodium distachyon BRI1* (*BRASSINOSTEROID INSENSITIVE 1*)-RNAi transgenic lines, as a BR-insensitive mutant, exhibited enhanced drought tolerance, accompanied by highly increased expression of several drought-responsive genes.

BR-signaling-related proteins, including BES1/BZR1 (BRI1 EMS SUPPRESSOR 1/BRASSINAZOLE RESISTANT 1), BIN2 (BRASSINOSTEROID INSENSITIVE 2), and BEH3 (BES1/BZR1 Homolog 3), can regulate ABA signaling output response in adaptation to dehydration stress [7,11]. Furthermore, Sun et al. [12] found that heterologous expression of *Zea mays BES1*/*BZR1*-*5* gene in transgenic *Arabidopsis thaliana* resulted in decreased ABA and drought sensitivity, implying that it positively mediates the ABA and abiotic stress responses. Additionally, BZR1 also positively regulates abiotic stress tolerance [13].

Proline is an important molecular chaperone that stabilizes protein structure, balances turgor pressure, attenuates dehydration, and regulates free radical levels [14,15]. We previously reported that the BEH3 protein of *A*. *thaliana* participates negatively in proline metabolism under dehydration stress conditions [7]. Moreover, our previous data showed that BEH3 is a negative regulator of BR-mediated hypocotyl length in dark-grown seedlings [7]. Therefore, BEH3 can coregulate BR signaling and dehydration stress response networks.

In this study, *GmBEH3L1*, an ortholog of *BEH3* in soybean (*Glycine max*), was isolated to examine the function of *GmBEH3L1* by heterologous expression in *A*. *thaliana*. *GmBEH3L1* was induced in response to ABA and water-deficit stress. The overexpression of *GmBEH3L1* in transgenic plants with the *beh3* mutant background was examined in response to ABA and osmotic stress. The complementary transgenic seedlings (*GmBEH3L1*-OE/*beh3*) appeared as ABA- and dehydration-stress-induced sensitive phenotypes in terms of cotyledon greening due to reduced proline contents, and limited the expression levels of stress-induced marker genes. Compared to osmotic-stress-treated *beh3* seedlings, *GmBEH3L1*-OE/*beh3* complementary lines display a sensitive phenotype in dehydration by regulating the expression levels of BR metabolite genes. These physiological experiments indicated similar mechanisms underlying the functions of *A*. *thaliana* BEH3 and *G. max* GmBEH3L1 in response to ABA and dehydration stress in *A*. *thaliana*. Thus, GmBEH3L1 is important in early seedling growth in response to ABA and water-deficit stress.

## 2. Results

### 2.1. Identification of Soybean BEH3 Orthologous Genes and Amino Acid Sequence Analysis

Recently, we reported a functional study of a nuclear-localized *BES1*/*BZR1* homolog gene, which has been designated BEH3 (for BES1/BZR1 Homolog 3), and described its physiological and molecular functions as a negative regulator of osmotic stress, linked to BR signaling in *A*. *thaliana* [7]. In this study, to further understand the function of the *BES1*/*BZR1* homolog gene in crops, we attempted to obtain the gene in *G. max* that encodes for amino acid sequences similar to the *A. thaliana* BEH3 protein. Overall, ten *BEH3-Like* genes from soybean were identified as a *BES1*/*BZR1* homolog gene family by BLAST search of the *G*. *max* genome (https://phytozome-next.jgi.doe.gov; *Glycine max* Wm82.a4.v1) (Figure S1). Among the ten BEH3 orthologs in soybean, two proteins, Glyma.12G231500 and Glyma.13G266500, show high amino acid sequence identity with *A. thaliana* BEH3 (Figures S1 and S2). Glyma.12G231500 and Glyma.13G266500 share 55% and 58% identity to *A. thaliana* BEH3, respectively (Figure S2). In addition, Glyma.12G231500 and Glyma.13G266500 contain a BES1-N-terminal active domain (BES1-ND) that is 84% and 82% identical, respectively, to the corresponding region of the *A*. *thaliana* BEH3 protein (Figure S1A). As shown in Figure S1B, a phylogenetic tree depicting distances between *A*. *thaliana BEH3* and *G*. *max BES1*/*BZR1* homologs was built with the clustering algorithm (MEGA-X program) [16].

### 2.2. GmBEH3L1 and GmBEH3L2 Are Upregulated by ABA and Dehydration Stress Treatments

**Figure 1:**
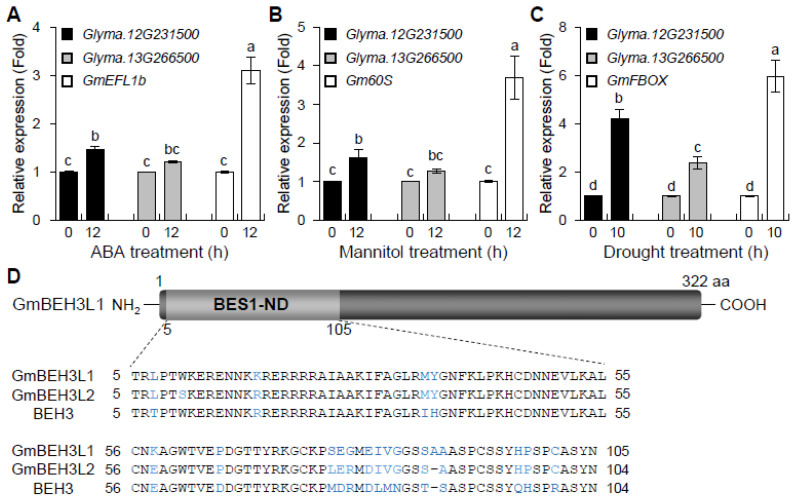
Expression analyses of *Glyma.12G231500* and *Glyma.13G266500* in soybean leaves treated with ABA or water deficit stresses and structural features of GmBEH3L1 protein. (**A**–**C**) *Glyma.12G231500* and *Glyma.13G266500* mRNA levels were determined by qPCR in soybean leaves. The leaves of 10-day-old seedlings treated with or without 100 μM ABA (**A**), 400 mM mannitol (**B**), and drought (**C**) stresses at indicated times were collected for expression analysis. *GmEFL1b*, *Gm60S*, and *GmFBOX* were used as controls for ABA or dehydration stress treatments. *Glycine max Actin 11* (*GmACT11*) (*Glyma.18G290800*) was used as the reference gene to normalize the qPCR analysis. Error bars represent the standard deviation of three independent biological replications. Lowercase letters indicate significant differences (*p* < 0.05) by Tukey’s multiple range test. (**D**) The primary structure of GmBEH3L1 harbors a BES1-ND motif region (5–105 amino acids), shown in a light gray box. Shown are the amino acid (aa) sequences for the BES1-ND motif in *Glycine max* BEH3-Like 1 (GmBEH3L1) (Glyma.12G231500), GmBEH3L2 (Glyma.13G266500) and *Arabidopsis thaliana* BEH3. Black and blue letters indicate identical and different amino acids, respectively. A gap (-) is introduced to optimize the aa sequences’ alignment.

To identify the relation between two soybean BEH3 orthologs (Glyma.12G231500 and Glyma.13G266500) and the ABA or dehydration response, the accumulation of these two mRNAs in 10-day-old soybean seedlings was estimated during ABA (100 μM), mannitol (400 mM), or drought treatment by quantitative real-time-PCR (qPCR). The qPCR data indicated that *Glyma.12G231500* transcripts showed 1.5-, 1.7- and 4.2-fold induction by ABA, osmotic stress, and drought treatment, respectively (Figure 1A–C). In contrast, *Glyma.13G266500* transcripts showed 1.2-, 1.2- and 2.4-fold induction by ABA, osmotic stress, and drought treatment, respectively (Figure 1A–C). Abiotic-stress-inducible *GmELF1b* (for *G*. *max Elongation Factor 1b*), *Gm60S* (for *G*. *max 60S*), and *GmFBOX* (for *G*. *max* F*-box protein*) [17] served as controls for the ABA, mannitol, and drought stress treatments, respectively (Figure 1A–C). These results demonstrate that *Glyma.12G231500* and *Glyma.13G266500* are regulated by ABA, dehydration, and drought stresses.

Meanwhile, the expression of *Glyma.12G231500* was more potently induced than that of *Glyma.13G266500* under ABA, osmotic stress and drought conditions (Figure 1A–C). Thus, *Glyma.12G231500* is a more essential biological gene involved in the regulation of abiotic stress responses. We therefore focused on the *Glyma.12G231500* gene in the next experiments, and named it *Glycine max BEH3-Like 1* (*GmBEH3L1*). Consequently, the *Glyma.13G266500* gene was designed as *GmBEH3L2*. The isolated full-length cDNA of *GmBEH3L1* was 969 bp and encoded 322 amino acids with a molecular weight of 34.8 kDa. The N-terminal sequence was well conserved in the 101 amino acid BES1 active motif (Figure 1D).

### 2.3. Overexpression of GmBEH3L1 Confers Sensitivity to ABA and Osmotic Stress Responses

**Figure 2:**
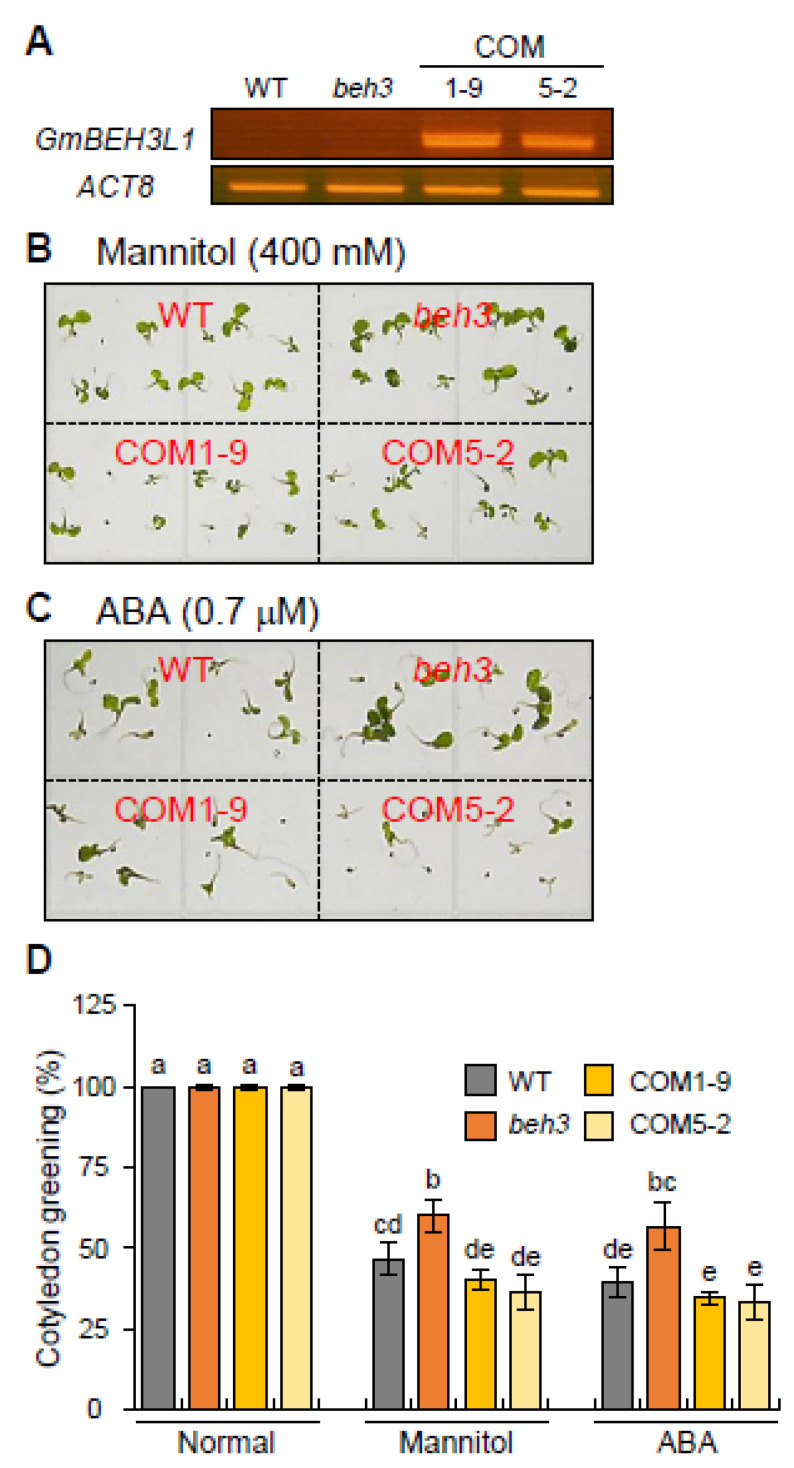
*GmBEH3L1*-overexpressing complementary (COM) lines reduced the cotyledon greening rates of *Arabidopsis thaliana* seedlings under osmotic stress and ABA condition. (**A**) Expression levels of *GmBEH3L1* in WT, *beh3*, and two individual *beh3*/ *GmBEH3L1*-overexpressing (COM1-9 and COM5-2) complementary transgenic lines were determined by RT-PCR using cDNAs generated from 10-day-old seedling mRNA. *Actin 8* (*ACT8*) was used in RT-PCR as a loading control. (**B**,**C**) Cotyledon greening assay under osmotic stress and ABA treatment. Cotyledon greening was photographed after 400 mM mannitol (**B**) and 0.7 μM ABA (**C**) treatments for 9 and 12 days, respectively. Cotyledon greening was defined as the proportion of cotyledons that had expanded and greened. (**D**) Analyses of cotyledon greening in complementary lines (COM1-9 and COM5-2) under the treatments of osmotic stress and ABA. The percentage of cotyledon greening was calculated for approximately 50 seedlings per replicate for each genotype at 9 and 12 days of growth on MS plates supplemented with 400 mM mannitol and 0.7 μM ABA, respectively. Error bars represent the standard deviation of three independent biological replications. Lowercase letters indicate significant differences (*p* < 0.05) by Tukey’s multiple range test.

To investigate whether *GmBEH3L1* was involved in the ABA and osmotic stress responses, we generated complementary transgenic lines, such as *GmBEH3L1*-overexpressing transgenic lines, in the *beh3* mutant background. For the overexpressed *GmBEH3L1* construct in a *beh3* mutant background (for complementary line; *GmBEH3L1*-OE/*beh3*), we obtained 10 homozygous independent complementary (COM) lines (T3 generation). Two of these lines were selected (COM1-9 and COM5-2), and the expression levels of *GmBEH3L1* were confirmed by reverse transcription (RT)-PCR and qPCR. The RT-PCR and qPCR results showed that *GmBEH3L1* was overexpressed in the *beh3* mutant (Figure 2A and Figure S3A). These two COM1-9 and COM5-2 lines were used to analyze abiotic-stress-responsive phenotypes.

To investigate the effects of *GmBEH3L1* expression on ABA or osmotic stress, seeds of wild-type (WT), *beh3*, and complementary lines (COM1-9 and COM5-2) were germinated on one-half-strength Murashige and Skoog (MS) plant medium. On MS medium (normal condition), the rate of seed germination or cotyledon greening was similar between WT and each genotype line (Figure S3B). On MS medium supplemented with 400 mM mannitol or 0.7 μM ABA, the rate of cotyledon greening of the *beh3* mutant was higher than WT and complementary plants, whereas two COM1-9 and COM5-2 complementary lines were slightly more sensitive to cotyledon greening than WT (Figure 2B–D). These physiological data indicated that the *GmBEH3L1*-expressing lines showed slightly or markedly more sensitivity toward dehydration or ABA than WT or *beh3*, respectively, during the early seedling growth stage (Figure 2B–D). Thus, the overexpression of *GmBEH3L1* in *beh3* is able to repress the dehydration- or ABA-insensitive traits of the *beh3* mutant.

### 2.4. Proline and Malondialdehyde (MDA) Contents of GmBEH3L1 Complementary Transgenic Lines under Dehydration Condition

**Figure 3:**
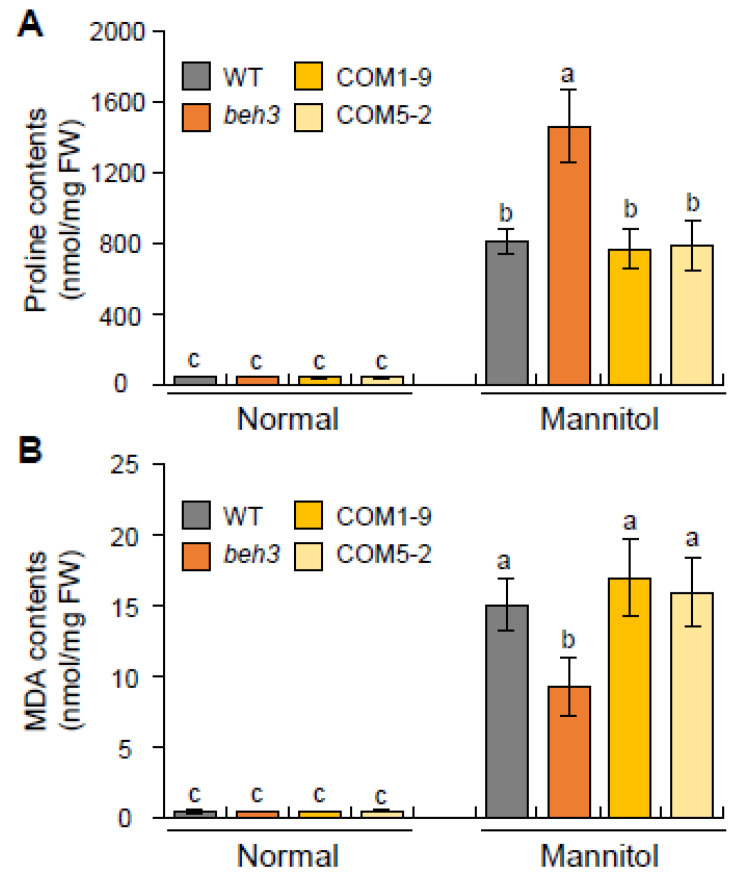
Analysis of dehydration-induced proline and MDA contents in seedlings of WT, *beh3*, and complementary transgenic plants. (**A**,**B**) Proline (**A**) and MDA (**B**) accumulation in 2-week-old seedling samples treated with or without (normal) 400 mM mannitol for 10 h. Error bars represent the standard deviation of three independent biological replications. Lowercase letters indicate significant differences (*p* < 0.05) by Tukey’s multiple range test.

To further analyze the response of *GmBEH3L1*-expressing complementary transgenic lines to osmotic stress, the proline or MDA content was measured. The proline or MDA content was more accumulated in WT, *beh3*, and complementary transgenic (COM1-9 and COM5-2) lines after a high concentration of mannitol treatment compared with no treatment (Figure 3). Among these samples, WT and the COM1-9 and COM5-2 lines showed significantly lower proline contents than the *beh3* mutant under the osmotic stress condition, whereas the accumulation of osmotic-stress-induced proline was similar between WT and complementary transgenic seedlings (Figure 3A). Moreover, the MDA contents of WT and complementary transgenic lines were higher than those of *beh3* under the osmotic stress condition (Figure 3B). Thus, GmBEH3L1 is necessary to control the proline and MDA contents in *beh3* under the osmotic stress condition. These results implied that the *GmBEH3L1*-expressing complementary transgenic seedlings displayed increased sensitivity to dehydration stress compared with the *beh3* mutant.

### 2.5. GmBEH3L1 Regulates the Expression of Osmotic-Stress-Related Genes under Dehydration Conditions

**Figure 4:**
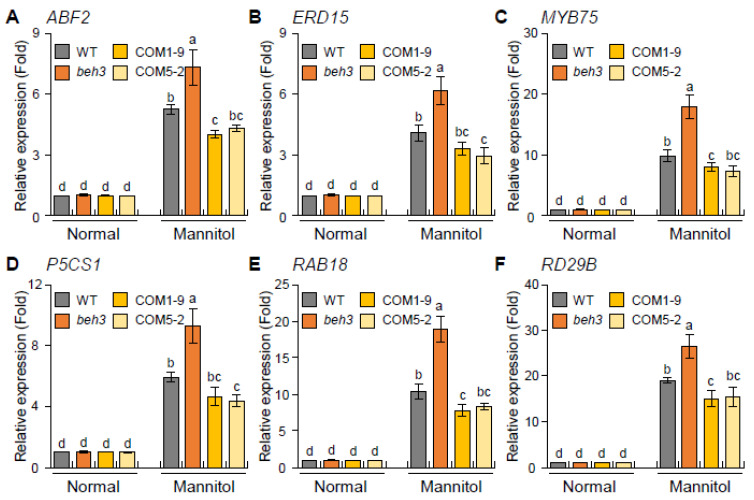
Expression patterns of dehydration-induced genes in response to osmotic stress. (**A**–**F**) The relative expression levels of dehydration-responsive *ABF2* (**A**), *ERD15* (**B**), *MYB75* (**C**), *P5CS1* (**D**), *RAB18* (**E**), and *RD29B* (**F**) genes were quantified by qPCR using mRNAs obtained from 12-day-old seedling samples supplemented with or without (normal) 400 mM mannitol. *ACT8* was used as the reference gene to normalize the qPCR analysis. Error bars represent the standard deviation of three independent biological replications. Lowercase letters indicate significant differences (*p* < 0.05) by Tukey’s multiple range test.

To observe whether the *GmBEH3L1*-overexpression construct influences the expression of osmotic-stress-inducible genes, including *ABF2* (for *Abscisic acid responsive elements-Binding Factor 2*), *ERD15* (for *Early Responsive to Dehydration 15*), *MYB75* (for *A. thaliana MYB 75*), *P5CS1* (for *delta 1-Pyrroline-5-Carboxylate Synthase 1*), *RAB18* (for *Responsive to ABA 18*), and *RD29B* (for *Responsive to Desiccation 29B*) [7,18,19,20,21], in COM1-9 and COM5-2 lines, we conducted a qPCR assay. Under the osmotic stress condition, the transcript levels of these six genes in *beh3* were higher than those in WT and two complementary seedlings, whereas the expression of these genes in complementary lines was decreased compared with WT seedlings (Figure 4). These data suggest that *GmBEH3L1* negatively regulates osmotic-stress-related genes.

### 2.6. GmBEH3L1 Negatively Regulates Hypocotyl Length in Response to Darkness by BR Signaling

Recently, we reported that the hypocotyl length of the *beh3* mutant in dark-grown seedlings was longer than in WT and *BEH3*-overexpressing transgenic seedlings [7]. In addition, we showed that BEH3 regulates the expression of BR metabolic-related genes under the dehydration condition. This means BEH3 negatively mediates hypocotyl elongation of early seedlings grown in darkness by regulating the expression of BR metabolic-responsive genes [7].

**Figure 5:**
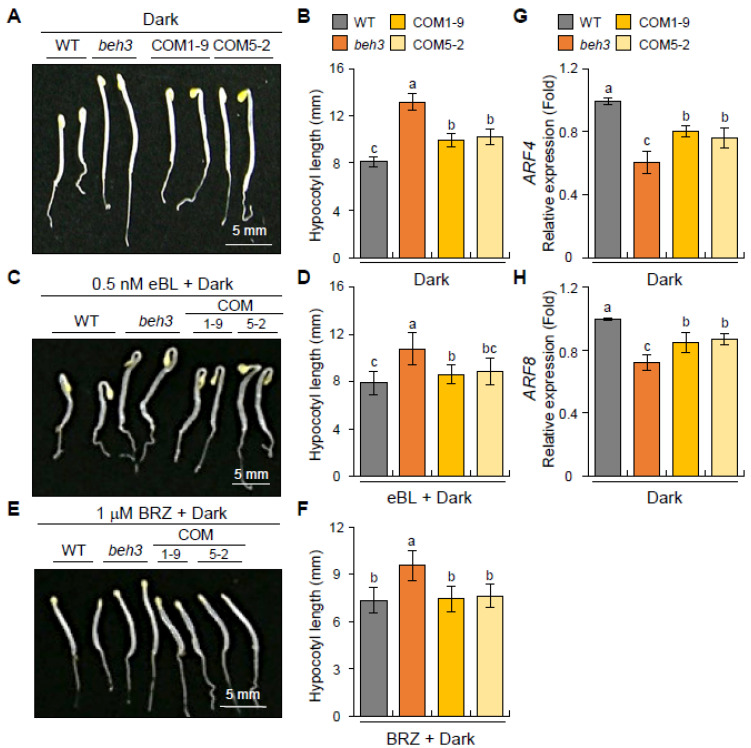
*GmBEH3L1*-expressing complementary lines modulate hypocotyl elongation in darkness**.** (**A**–**F**) Hypocotyl elongation response of 3-day-old seedlings in darkness. Seeds of WT, *beh3*, and *GmBEH3L1*-expressing complementary lines were sown in plant growth medium supplemented without (**A**,**B**) and with 0.5 nM eBL (**C**,**D**) or 1 μM BRZ (**E**,**F**) and allowed to grow vertically for 3 days in darkness condition. Error bars represent the standard deviation of three independent biological replications; for each replicate, at least 10 individual seedlings were calculated per sample. Lowercase letters indicate significant differences (*p* < 0.05) by Tukey’s multiple range test. Scale bars = 5 mm. (**G**,**H**) Expression patterns of hypocotyl elongation responsive genes in response to darkness. The relative expression levels of *ARF4* (**G**) and *ARF8* (**H**) genes were quantified by qPCR using mRNAs obtained from 3-day-old dark-grown seedling samples. Error bars represent the standard deviation of three independent biological replications. Lowercase letters indicate significant differences (*p* < 0.05) by Tukey’s multiple range test.

To investigate the effects of *GmBEH3L1* expression on hypocotyl elongation in dark-grown seedlings, the seeds of WT, *beh3*, and *GmBEH3L1*-expressing complementary transgenic lines (COM1-9 and COM5-2) were germinated on one-half-strength MS plant medium in the dark. The hypocotyl length of each genotype seedling was calculated 3 days after germinating in the dark-grown condition. For dark-grown seedlings, the hypocotyl lengths of complementary lines (COM1-9 and COM5-2) were longer than that of WT but shorter than that of *beh3* seedlings (Figure 5A,B). This result implied that overexpression of *GmBEH3L1* caused the short hypocotyl phenotype during growth in the dark compared with the *beh3* mutant.

To further analyze *GmBEH3L1* expression in *A*. *thaliana* in response to BR, we measured the hypocotyl length of dark-grown seedlings after 24-epibrassinolide (eBL) or brassinazole (BRZ) treatment. In response to 0.5 nM eBL under dark conditions, the hypocotyl lengths of COM1-9 and COM5-2 seedlings were slightly longer than those of WT seedlings but shorter than those of *beh3* seedlings (Figure 5C,D). Thus, eBL partially rescued the BR-induced hypocotyl length defects of *GmBEH3L1*-expressing transgenic seedlings under dark conditions.

Subsequently, we measured the hypocotyl lengths of each dark-grown genotype seedling after BRZ (BR-synthesis inhibitor) treatment. After treatment with 1 μM BRZ, complementary transgenic lines (COM1-9 and COM5-2) showed shorter hypocotyl lengths than *beh3* seedlings, whereas the hypocotyl lengths were similar among WT and the two complementary transgenic seedlings (Figure 5E,F), suggesting that *GmBEH3L1*-expressing transgenic seedlings exhibited relatively weak BR-deficient traits compared with the *beh3* mutant.

Additionally, to investigate whether GmBEH3L1 could be involved in the regulation of hypocotyl length under darkness, we performed qPCR to analyze the expression levels of *ARF4* (for *Auxin Response Factor 4*) and *ARF8*, which are related to hypocotyl elongation [22]. Under dark conditions, the transcript levels of these two genes in two complementary lines were lower than those in WT plants, whereas they were higher in COM1-9 and COM5-2 lines than in the *beh3* mutant (Figure 5G,H). These data suggest that GmBEH3L1 regulates these hypocotyl elongation-responsive genes in darkness. Collectively, these hypocotyl elongation experiments, as shown in Figure 5, showed that GmBEH3L1 plays an important role in mediating hypocotyl elongation by BR signaling in darkness.

### 2.7. GmBEH3L1 Regulates the Expression of BR-Metabolite Genes under Dehydration Conditions

**Figure 6:**
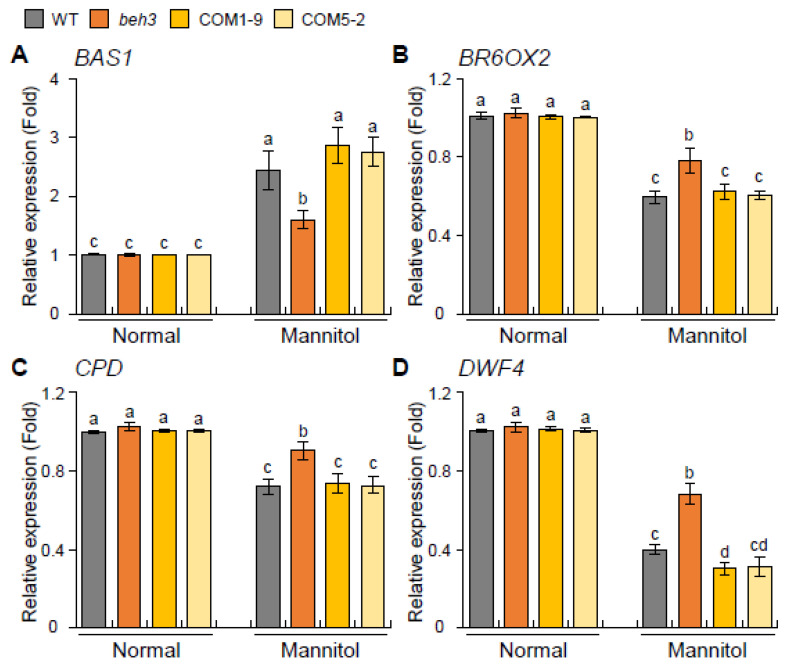
Expression patterns of BR-metabolite-responsive genes in response to osmotic stress. (**A**–**D**) The relative expression levels of BR-metabolite-responsive *BAS1* (**A**), *BR6OX2* (**B**), *CPD* (**C**), and *DWF4* (**D**) genes were quantified by qPCR using mRNAs obtained from 12-day-old seedling samples treated with or without (normal) 400 mM mannitol. *ACT8* was used as the reference gene to normalize the qPCR analysis. Error bars represent the standard deviation of three independent biological replications. Lowercase letters indicate significant differences (*p* < 0.05) by Tukey’s multiple range test.

To investigate the BR-metabolic genes associated with dehydration stress in *GmBEH3L1*-expressing transgenic lines, we chose to analyze the expression levels of four genes *BAS1* (for *PhyB-4 Activation-tagged Suppressor 1*), *BR6OX2* (for *Brassinosteroid-6-Oxidase 2*), *CPD* (for *Constitutive Photomorphogenic Dwarf*), and *DWF4* (for *Dwarf 4*), which are regulated by several abiotic stresses [7,23]. Under the normal condition, the transcript levels of these four genes in *beh3* and two complementary lines were not much different from WT. Under the dehydration stress condition, the transcript level of BR-catabolic *BAS1* was lower in the *beh3* mutant than in the complementary lines (COM1-9 and COM5-2), which were similar to the expression levels in WT (Figure 6A). As shown in Figure 6B–D, the expression levels of BR-biosynthesis *BR6OX2*, *CPD*, and *DWF4* genes were higher in the *beh3* mutant than in WT and complementary lines under the osmotic stress condition. Although the expression level of *DWF4* was slightly lower in the complementary lines than in WT under the dehydration condition, the expression levels of BR-biosynthesis genes *BR6OX2* and *CPD* were similar between WT and complementary lines after the osmotic stress treatment (Figure 6B–D). These qPCR results show that GmBEH3L1 can regulate the expression of these BR-metabolic genes in seedlings during growth under dehydration conditions.

## 3. Discussion

To identify a gene associated with the dehydration stress response in *G. max*, we isolated the *A*. *thaliana BEH3* ortholog gene, *GmBEH3L1* (*Glyma.12G231500*) (Figure 1 and Figure S1), for functional characterization. The GmBEH3L1 protein contains a single BES1-ND domain in its N-terminal region (Figure 1D), and nine BES1-ND-harboring proteins in the soybean genome show high amino acid sequence similarity to the GmBEH3L1 protein (Figures S1 and S2). Based on the amino acid sequence analysis, GmBEH3L2 also harbors a conserved BES1-ND motif (Figure 1D and Figure S1), which is upregulated by ABA and dehydration stress (Figure 1A–C). As reported previously by Yin et al. [24], the BES1-ND domain was required for protein activity and turnover in the BR signaling pathway, indicating that BES1-ND-harboring proteins are involved in the BR and abiotic stress responses.

Considering the high identity between the *A*. *thaliana* BEH3 protein and soybean GmBEH3L1 protein (Figure 1D and Figure S2), we wondered whether BEH3 and GmBEH3L1 would exhibit a similar function in response to ABA and dehydration stress. As shown in Figure 2, complementary lines (*GmBEH3L1*-OE/*beh3*) were slightly more sensitive to ABA or dehydration stress than WT, whereas insensitivity to ABA or dehydration stress was confirmed in the *beh3* mutant. In addition, the overexpression of *GmBEH3L1* in *beh3* was able to suppress the ABA- or dehydration-insensitive trait of the *beh3* mutant (Figure 2). These results indicate that the physiological functions of the soybean GmBEH3L1 protein are similar to those of the *A*. *thaliana* BEH3 protein in response to ABA and osmotic stress.

Proline is an important compatible solute in plant responses to osmotic stress. It has many protective roles, including balancing turgor pressure, regulating antioxidant production, stabilizing protein structure, protecting cellular membrane structure, and attenuating dehydration during plant abiotic stress [7,15,25]. Furthermore, ABA and BR phytohormones also mediate proline accumulation to confer tolerance to various abiotic stresses [7,26,27]. In the present study, the proline contents in WT and complementary seedlings were lower than those of *beh3* under the osmotic stress condition, whereas the MDA contents of WT and *GmBEH3L1*-expressing transgenic lines were higher than those of the *beh3* mutant in response to dehydration (Figure 3), indicating that the overexpression of *GmBEH3L1* in *beh3* resulted in cell damage induced by lipid peroxidation. These physiological data suggest that GmBEH3L1 regulates proline accumulation geared toward protecting the cellular membrane structure under osmotic stress. Thus, GmBEH3L1 may negatively mediate the dehydration response during the early seedling stage.

In our study, the mRNA levels of osmotic-stress-inducible genes, including *ABF2*, *ERD15*, *MYB75*, *P5CS1*, *RAB18*, and *RD29B* [28,29,30], were estimated by a qPCR assay (Figure 4). The levels were lower in *GmBEH3L1*-expressing transgenic lines than in WT and *beh3* seedlings (Figure 4), which implies that GmBEH3L1 could regulate the signal necessary for mediating the expression of osmotic-stress-inducible genes.

Based on phenotypic and genetic experiments, BR signaling or endogenous BR concentration regulates hypocotyl elongation, abiotic stress tolerance, and reactive oxygen species accumulation [7,31,32,33]. We previously showed that 3-day-old *beh3* seedlings grown in darkness revealed markedly longer hypocotyls than WT seedlings, whereas hypocotyl lengths of *BEH3*-overexpressing seedlings were shorter than WT seedlings, also grown in the dark [7]. As shown in Figure 5A,B, the hypocotyl lengths of WT and *GmBEH3L1*-OE/*beh3* complementary lines (COM1-9 and COM5-2) were shorter than that of *beh3* seedlings, whereas complementary lines displayed longer hypocotyl lengths than WT seedlings, all under dark conditions. Moreover, complementary lines displayed higher mRNA levels of hypocotyl-length-regulatory genes (*ARF4* and *ARF8*) under dark conditions compared with *beh3* plants, also grown in darkness (Figure 5G,H). Taken together, these findings indicate that GmBEH3L1 may negatively regulate hypocotyl length in seedlings grown in darkness. Thus, these phenotypic and molecular data suggest similar genetic functions between soybean GmBEH3L1 and the *A*. *thaliana* BEH3 protein in dark-induced signaling.

Under the osmotic stress condition, the expression levels of BR-synthesis-related genes *BR6OX2*, *CPD*, and *DWF4* were lower in complementary lines than in the *beh3* mutant but similar between WT and complementary seedlings, except for the *DWF4* gene, which was decreased more in complementary lines compared with WT (Figure 6). Additionally, the expression of the BR-catabolic *BAS1* gene was higher in complementary lines than in *beh3* plants (Figure 6A). These observations demonstrate that GmBEH3L1 might regulate the endogenous BR level in the early seedling stage under osmotic stress conditions. Consequently, the hypocotyl elongations of BR-treated complementary seedlings partially rescued the phenotype of *beh3* seedlings grown in the dark condition (Figure 5C,D), implying that GmBEH3L1 acts as a negative regulator of hypocotyl length of dark-grown seedlings by mediating the endogenous BR level.

In conclusion, our study provides evidence for a similar mechanism of action of the *A*. *thaliana* BEH3 protein and soybean GmBEH3L1 protein in regulating osmotic stress-induced sensitive parameters, including cotyledon greening rate, proline accumulation, MDA content, and transcript levels of stress-related marker genes in *A*. *thaliana* exposed to osmotic stress. Thus, our finding describes the possibility that GmBEH3L1 controls the dehydration response in soybean, similar to the BEH3 mechanism. In addition, *GmBEH3L1*-OE/*beh3* complementary transgenic lines suppress a long hypocotyl trait of the *beh3* mutant grown in darkness, implying the existence of similar roles between soybean GmBEH3L1 and the *A*. *thaliana* BEH3 protein in the BR signaling pathway. Collectively, our results demonstrate that GmBEH3L1 is a negative regulator of the dehydration stress response and the BR signaling pathways.

## 4. Materials and methods

### 4.1. Plant Growth Conditions and Abiotic Stress Treatments

*A*. *thaliana* ecotype Columbia-0 (Col-0), *beh3*, *GmBEH3L1*-expressing transgenic lines, and soybean [*G. max* (L.) Merr.] plants were grown in a controlled growth room (16 h light/8 h night cycle, 60% relative humidity, 24 ± 2 °C, and light intensity of 140 μmol m^−2^ s^−1^). For dehydration stress or ABA responses, excised leaves of 10-day-old soybean seedlings were submerged and shaken in a solution containing 400 mM mannitol or 100 μM ABA. Samples were obtained at 0 and 12 h after treatment with mannitol or ABA. For drought response, the leaves were excised from 10-day-old soybean seedlings and placed in a Petri dish in a growth incubator (28 ± 2 °C). Samples were obtained at 0 and 10 h after the drought treatment. In each case, obtained samples were frozen in liquid nitrogen promptly for total RNA extraction.

### 4.2. Extraction of Total RNA and RT-PCR and qPCR Analyses

Total RNA was extracted with a Plant RNeasy extraction kit (Qiagen, Valencia, CA, USA). cDNA was synthesized with 3 μg of total RNA using the RevertAid First Strand cDNA Synthesis Kit (Fermentas, Burlington, ON, Canada). RT-PCR proceeded for 30 cycles as follows: 94 °C, 15 s; 57 °C, 15 s; 72 °C, 1 min. qPCR was performed with a CFX Connect quantitative PCR machine (Bio-Rad, Hercules, CA, USA). The iQ SYBR Green Supermix kit (Bio-Rad) was used for qPCR analysis. The reaction primers used in RT-PCR or qPCR are listed in Table S1.

### 4.3. Overexpression Construct of GmBEH3L1

To generate *GmBEH3L1*-overexpressing transgenic lines in the *beh3* mutant background (*GmBEH3L1*-OE/*beh3*), full-length *GmBEH3L1* cDNA was amplified using gene-specific primers (Table S1). The generated cDNA was cloned into the pDONR/ZEO vector and verified by sequencing. This plasmid was then sub-cloned into the pGWB514 plant constitutive expression vector under the control of the CaMV 35S promoter by using a Gateway system (Invitrogen, Carlsbad, CA, USA). The *GmBEH3L1*-overexpression construct was transformed into the *beh3* mutant using *Agrobacterium tumefaciens* strain GV3101 through in planta vacuum infiltration. Hygromycin-resistant T2 transformants were segregated as a single locus. T3 or T4 homozygous *GmBEH3L1*-expressing transgenic (*GmBEH3L1*-OE/*beh3*) plants were used for phenotypic and molecular characterization.

### 4.4. Phenotype Analyses under ABA or Dehydration Stress Treatment

To analyze the response to ABA or dehydration stress, seeds were sown on MS medium supplemented with 0.7 μM ABA or 400 mM mannitol. The cotyledon greening of seedlings was recorded at 9–12 days after seed germination. Cotyledon greening was defined as the proportion of cotyledons that had expanded and greened. For hypocotyl elongation analysis, seeds were sown on MS medium supplemented with 1 μM BRZ or 0.5 nM eBL under dark conditions. The test was performed three times with at least 10 seedlings per sample per replicate.

### 4.5. Determination of Proline and MDA Contents

Proline contents were determined as previously described by Bates et al. [34]. Proline was extracted from 150 mg of seedling leaves in 3% sulfosalicylic acid. Afterward, 200 μL of the extract was added to 100 μL of ninhydrin buffer (6.8% phosphoric acid, 80% glacial acetic acid, and 70.17 mM ninhydrin) and then boiled at 95 °C for 1 h. The reaction mixture was cooled by placing it on an ice bath. Once cooled, the reaction mixture was added to 200 μL toluene and lightly swirled. The absorbance of the toluene layer was measured at 520 nm using a UV/VIS spectrophotometer (JASCO, Tokyo, Japan). Proline concentration was extrapolated based on a standard curve and estimated on a fresh weight (FW) basis as follows: [(ng proline/mL × mL extraction solution)/115.5 ng nmol]/g sample = nmol proline/g FW material.

MDA contents were determined by the thiobarbituric acid (TBA) chemical reaction [29]. Leaf samples (2–4 g) were homogenized in 2–4 mL of 0.1% trichloroacetic acid and centrifuged at 10,280× *g* for 20 min. Then, 2–4 mL of the supernatant was added to 1 mL of 0.6% TBA, heated at 90 °C for 20 min, cooled rapidly on ice, and centrifuged at 2570× *g* for 20 min. The absorbance of the colored supernatant was measured at 532 nm, and the non-specific absorbance at 450 and 600 nm was subtracted.

### 4.6. Statistical Analysis

Statistical analysis, including one-way analysis of variance and Tukey’s multiple range test, was performed with SPSS 23.0 software program (IBM Corp., Armonk, NY, USA). Different letters on the graphs indicate statistically significant differences at *p* < 0.05.

## Data Availability

Not applicable.

## Supplementary Materials

The following supporting information can be downloaded at: https://www.mdpi.com/article/10.3390/plants11192565/s1, Figure S1: Analysis of amino acid sequences of BES1-ND domain in BES1/BZR1 family homologous genes and phylogenetic relationship of BEH3 orthologs from soybean (*Glycine max*); Figure S2: Alignment of amino acid sequences of two soybean BEH3 orthologs (Glyma.12G231500 and Glyma.13G266500) and *Arabidopsis thaliana* BEH3; Figure S3: Genotype analysis of *GmBEH3L1*-expressing complementary lines; Table S1: Primers used for RT-PCR, qPCR, and gene cloning.
