# Supplementary material for: Isolation and Functional Characterization of Soybean BES1/BZR1 Homolog 3-Like 1 (GmBEH3L1) Associated with Dehydration Sensitivity and Brassinosteroid Signaling in Arabidopsis thaliana
Source: Plants (Basel). 2022 Sep 29;11(19):2565. doi: 10.3390/plants11192565 (PMC9573144; doi:10.3390/plants11192565)
Supplement: Supplementary file 1 [file plants-11-02565-s001.zip › plants-1884643-supplementary.pdf]

Supplementary Materials

Table S1. Primers used for qPCR, RT-PCR and gene cloning

qPCR and RT-PCR	Primer sequence (5' to 3')
GmBEH3L1 (*Glyma_12G231500*)	Forward: TCAGAGGGCATGGAGATAGT<br>Reverse: GAGTCCACATTGGCGAACCG
Glyma_13G266500	Forward: CCACATTCTTCTTATAACAC<br>Reverse: GTTGTTTTCTCTCTCCTTCGA
GmACT11 *(Glyma_18G290800)*	Forward: ATCTTGACTGAGCGTGGTTATTCC<br>Reverse: GCTGGTCCTGGCTGTCTCC
GmEFL1b (*Glyma_02G276600*)	Forward: ATGGCTGTCACCTTCTCAGA<br>Reverse: CATATCCCACAGGAACCAGT
Gm60S (*Glyma_13G318800*)	Forward: TCAACCGTATACCCCGGACA<br>Reverse: CTTCACTGCCTCCTTCAGCA
GmFBOX (*Glyma_12G051100*)	Forward: ACCAGGTCTCCTCTTACCGAA<br>Reverse: TGAGGTTCAAGGGACAAGCG
ACT8 (*At1g49240*)	Forward: CCTTGCTGGTCGTGACCTTACTGA<br>Reverse: CTCTCAGCACCGATCGTGATCACT
ABF2 (*At1g45249*)	Forward: GAGGTCAAGAGGGTTTGCAG<br>Reverse: GAATTTGCAGTCTCTGCACC
ERD15 (*At2g41430*)	Forward: CCAGCGAAATGGGGAAACCA<br>Reverse: ACAAAGGTACAGTGGTGGC
MYB75 (*At1g56650*)	Forward: AGTATGGAGAAGGCAAATGG<br>Reverse: GTAGGAATGGGCGTAATGTC
P5CS1 (*At2g39800*)	Forward: CGACGGAGACAATGGAATTGT<br>Reverse: GATCAGAAATGTGTAGGTAGC
RAB18 (*At5g66400*)	Forward: CGATCCAGCAGCAGTATGAC<br>Reverse: TTCGAAGCTTAACGGCCACC
RD29B (*At5g52300*)	Forward: CGTCCTTATGGTCATGAGC<br>Reverse: GCCTCATGTCCGTAAGAGG

ARF4 (*At5g60450*)	Forward: GCACACATGGAGGCTTCTCT<br>Reverse: ACCGAGGGATCAATCTCCCA
ARF8 (*At5g37020*)	Forward: GCAGCAGCAATCAGAGATGC<br>Reverse: ATTCTGCGGATTTCCGGGTT
BAS1 (*At2g26710*)	Forward: GTAGCAGCTGGTTCATTCCA<br>Reverse: CAGGATCGGCTACCGTTAAC
BR6OX2 (*At3g30180*)	Forward: CAACATAGCTGCGGTTCATG<br>Reverse: GTAACCCAACATGTCTGTGA
CPD (*At5g05690*)	Forward: CGAAGATCGGGTACCGTTTCTG<br>Reverse: CAAAGATGCTCGCACTTTCAAC
DWF4 (*At3g50660*)	Forward: GCTGAGATCACCGGTAACAC<br>Reverse: CTCTCGCTATCTTCTTCTTGC
**Gene cloning**	**Primer sequence (5' to 3')**
GmBEH3L1 (*Glyma_12G231500*) for complementary transgenic line	Forward: GGGGACAAGTTTGTACAAAAAAGCAGGCTTCAT GACGTCGGGGACGAGGCT<br>Reverse: GGGGACCACTTTGTACAAGAAAGCTGGGTCTCAT CTGGTCTTTGAGTTAC

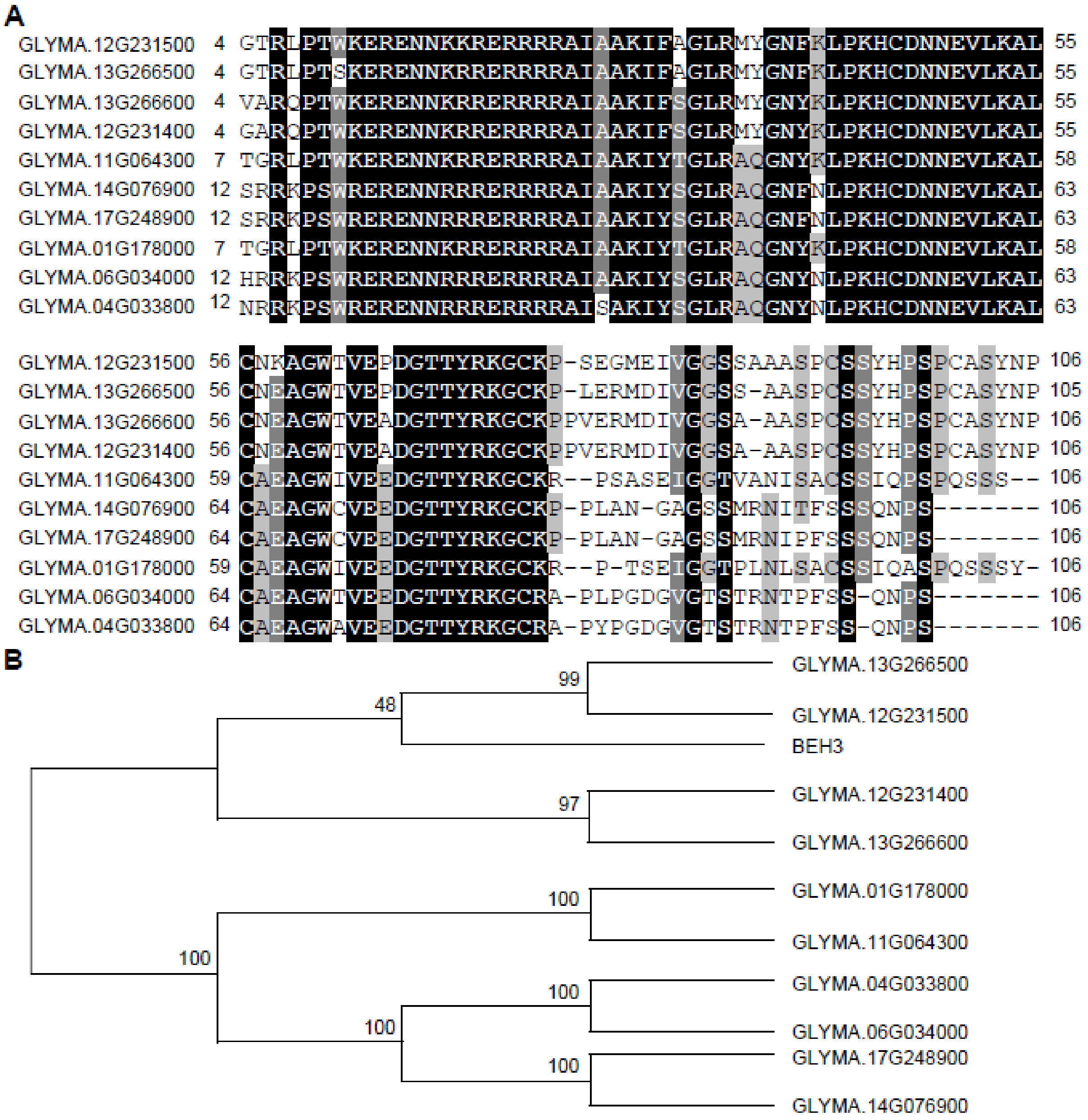

Figure S1. Analysis of amino acid sequences of BES1-ND domain in BES1/BZR1 family homologous genes and phylogenetic relationship of BEH3 orthologs from soybean (*Glycine max*). (**A**) Alignment of BES1-ND domain sequences of GLYMA_12G231500 and other BES1/BZR1 homolog genes from *G. max* species. Shown are the BES1-ND domain sequences of GLYMA_12G231500, GLYMA_13G266500, GLYMA_13G266600, GLYMA_12G231400, GLYMA_11G064300, GLYMA_14G076900, GLYMA_17G248900, GLYMA_01G178000, GLYMA_06G034000, and GLYMA_04G033800 (https://phytozome-next.jgi.doe.gov). Gray and black shading describe similar and identical amino acids, respectively. Gap (-) is introduced to optimize the amino acid sequence alignment. (**B**) Phylogenetic relationship of BEH3 orthologs from soybean (*G. max*). The bootstrap values are

shown near the corresponding branch (MEGA-X program).

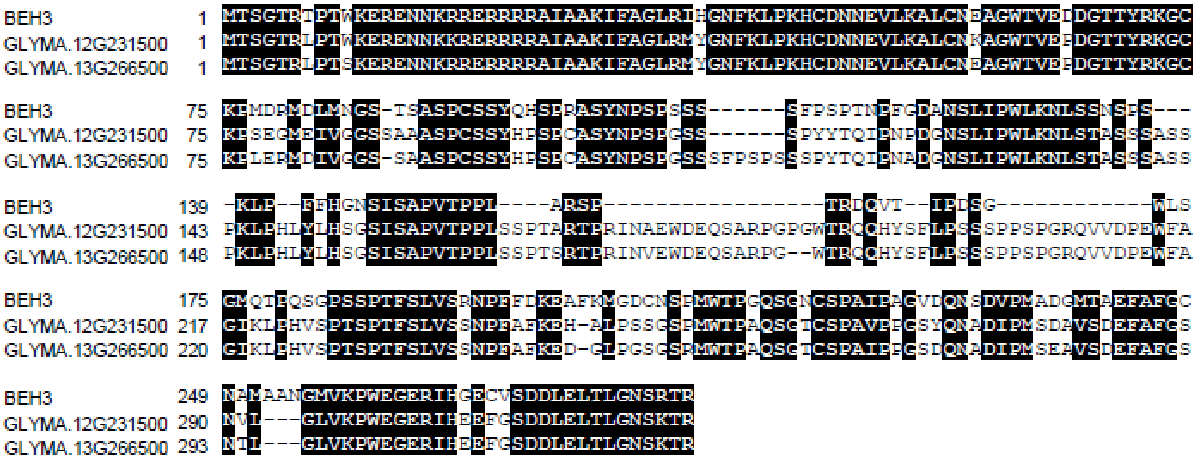

Figure S2. Alignment of amino acid sequences of two soybean BEH3 orthologs (GLYMA_12G231500 and GLYMA_13G266500) and *Arabidopsis thaliana* BEH3. Shown are the amino acid sequences of *Glycine max* GLYMA_12G231500, GLYMA_13G266500, and *A. thaliana* BEH3 (At4g18890). Black shading describes identical amino acids. Gap (-) is introduced to optimize the amino acid sequence alignment.

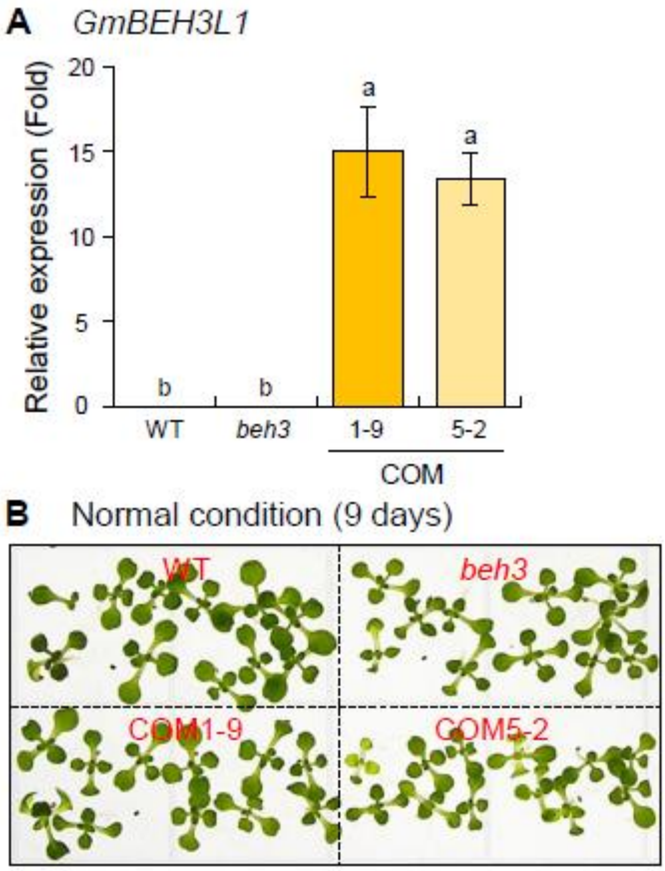

Figure S3. Genotype analysis of *GmBEH3L1*-expressing complementary lines. (**A**) Genotype analysis of WT, *beh3*, and two individual *GmBEH3L1*-expressing complementary (COM1-9 and COM5-2) seedlings. The expression level of the *GmBEH3L1* gene was quantified by qPCR using mRNAs obtained from 10-day-old seedling samples. Error bars represent the standard deviation of three independent biological replications. Lowercase letters indicate significant differences ($P < 0.05$) by Tukey's multiple range test. (**B**) Photos of WT, *beh3*, and *GmBEH3L1*-expressing complementary (COM1-9 and COM5-2) seedlings at 9 days under the normal condition.
